# Performance of a deep learning tool to detect missed aortic dilatation in a large chest CT cohort

**DOI:** 10.3389/fcvm.2022.972512

**Published:** 2022-08-22

**Authors:** Maurice Pradella, Rita Achermann, Jonathan I. Sperl, Rainer Kärgel, Saikiran Rapaka, Joshy Cyriac, Shan Yang, Gregor Sommer, Bram Stieltjes, Jens Bremerich, Philipp Brantner, Alexander W. Sauter

**Affiliations:** ^1^Department of Radiology, Clinic of Radiology & Nuclear Medicine, University Hospital Basel, University of Basel, Basel, Switzerland; ^2^Department of Radiology, Northwestern University Feinberg School of Medicine, Chicago, IL, United States; ^3^Siemens Healthineers, Forchheim, Germany; ^4^Siemens Healthineers, Princeton, NJ, United States; ^5^Hirslanden Klinik St. Anna, Luzern, Switzerland; ^6^Regional Hospitals Rheinfelden and Laufenburg, Rheinfelden, Switzerland; ^7^Department of Radiology, University Hospital Tuebingen, University of Tuebingen, Tuebingen, Germany

**Keywords:** aorta - thoracic, aortic aneurysm (thoracic), deep learning, dilatation, computed tomography, guidelines, diameter measurement, artifical intelligence (AI)

## Abstract

**Purpose:**

Thoracic aortic (TA) dilatation (TAD) is a risk factor for acute aortic syndrome and must therefore be reported in every CT report. However, the complex anatomy of the thoracic aorta impedes TAD detection. We investigated the performance of a deep learning (DL) prototype as a secondary reading tool built to measure TA diameters in a large-scale cohort.

**Material and methods:**

Consecutive contrast-enhanced (CE) and non-CE chest CT exams with “normal” TA diameters according to their radiology reports were included. The DL-prototype (AIRad, Siemens Healthineers, Germany) measured the TA at nine locations according to AHA guidelines. Dilatation was defined as >45 mm at aortic sinus, sinotubular junction (STJ), ascending aorta (AA) and proximal arch and >40 mm from mid arch to abdominal aorta. A cardiovascular radiologist reviewed all cases with TAD according to AIRad. Multivariable logistic regression (MLR) was used to identify factors (demographics and scan parameters) associated with TAD classification by AIRad.

**Results:**

18,243 CT scans (45.7% female) were successfully analyzed by AIRad. Mean age was 62.3 ± 15.9 years and 12,092 (66.3%) were CE scans. AIRad confirmed normal diameters in 17,239 exams (94.5%) and reported TAD in 1,004/18,243 exams (5.5%). Review confirmed TAD classification in 452/1,004 exams (45.0%, 2.5% total), 552 cases were false-positive but identification was easily possible using visual outputs by AIRad. MLR revealed that the following factors were significantly associated with correct TAD classification by AIRad: TAD reported at AA [odds ratio (OR): 1.12, *p* < 0.001] and STJ (OR: 1.09, *p* = 0.002), TAD found at >1 location (OR: 1.42, *p* = 0.008), in CE exams (OR: 2.1–3.1, *p* < 0.05), men (OR: 2.4, *p* = 0.003) and patients presenting with higher BMI (OR: 1.05, *p* = 0.01). Overall, 17,691/18,243 (97.0%) exams were correctly classified.

**Conclusions:**

AIRad correctly assessed the presence or absence of TAD in 17,691 exams (97%), including 452 cases with previously missed TAD independent from contrast protocol. These findings suggest its usefulness as a secondary reading tool by improving report quality and efficiency.

## Introduction

Dilatation of the thoracic aorta can lead to aortic aneurysms and ultimately death; thus, more 6,000 people died of aortic aneurysms in the US in 2020 (1–3). Imaging allows diagnosis of thoracic aortic dilatation (TAD) and current guidelines require measurements perpendicular to the blood flow axis for adequate diameter measurements (2, 4). Those measurements are typically performed on ECG-triggered CT angiography when TAD was initially suspected (2, 4, 5). In other scenarios, they are not systematically performed in clinical routine. The candy-cane shape of the thoracic aorta, different scan protocols and an overall increased workload may prevent TAD from being diagnosed (6–9). The rate of missed TAD by radiologists has not yet been assessed in a larger cohort.

Deep learning (DL) is an advanced artificial intelligence (AI) technique. Recently, it was successfully applied to perform guideline-compliant diameter measurements of the thoracic aorta in dedicated ECG-triggered CT exams in two small cohorts (10, 11). In this study, we applied this prototype software [AI-Rad Companion (AIRad)] to a large data set of more than 18,000 chest CT exams. In all those exams, which included varying imaging protocols, aortic diameters were previously reported as “normal” in the corresponding reports. Aim of this study was to evaluate the performance of AIRad as a secondary reading tool to detect missed TAD. Furthermore, we investigated imaging- and patient-based parameters associated with correct TAD classification by AIRad.

## Materials and methods

### Study cohort

**Figure 1:**
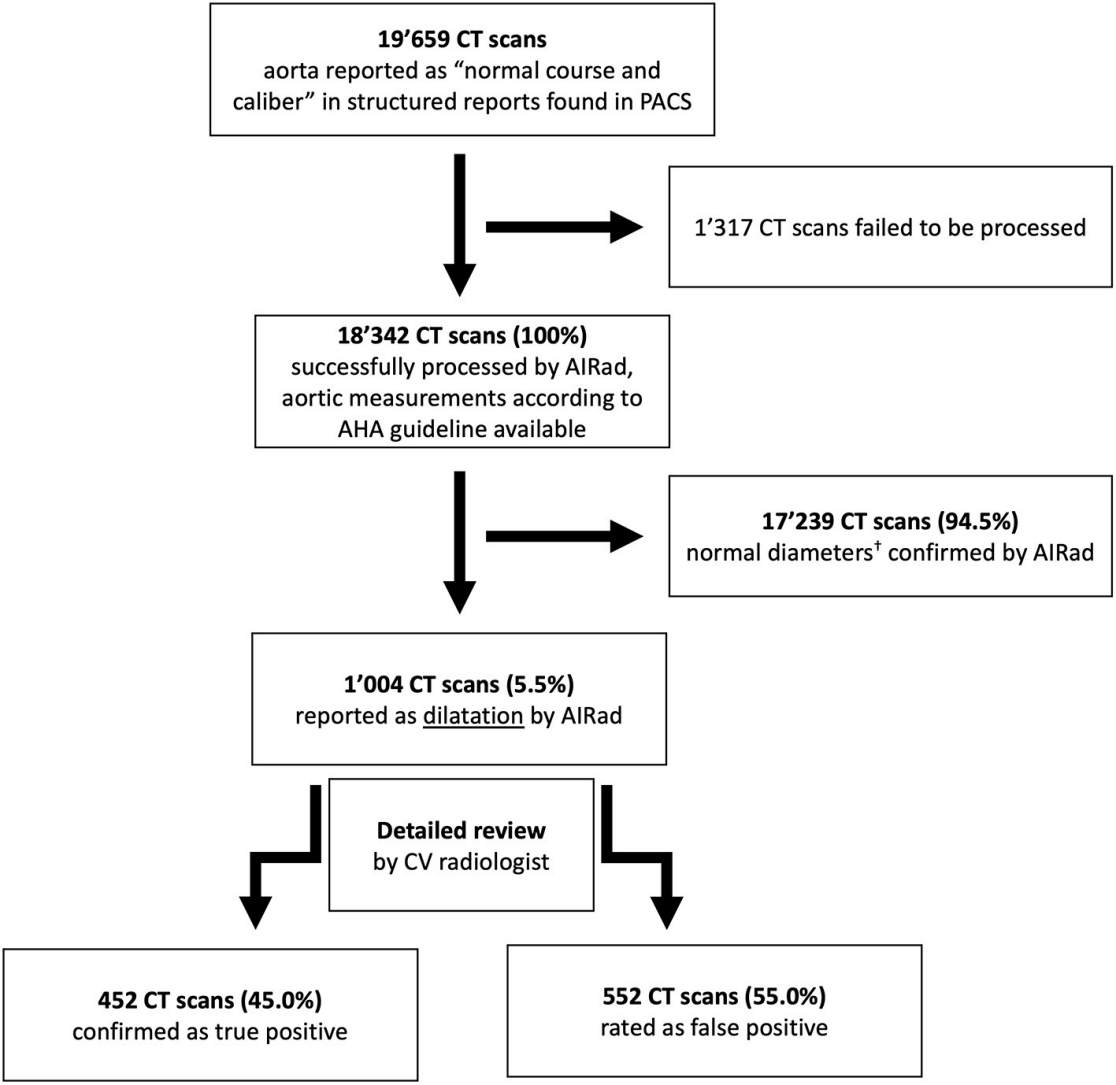
Flow chart. ^†^ <45 mm for aortic sinus, sinotubular junction, ascending aorta and proximal arch or <40 mm at mid and distal arch, mid and distal descending aorta and abdominal aorta. AHA, American Heart Association; CV, cardiovascular.

This retrospective study was approved by the local Ethics Committee (Ethikkommission Nordwest- und Zentralschweiz, ID: 2019-01053), the need for informed consent was waived. Figure 1 shows the study flow chart. First, we searched our PACS for all chest CT exams using an in-house developed PACS Crawler (12, 13). In a second step, we identified all exams with structured reports available between 01/2016 (when standard reports were introduced at our institution) and 06/2019. Finally, we selected only exams in which the aorta was reported as “normal course and caliber” (standard phrase) and approved by a board-certified radiologist. A total of 19,659 exams were identified for this study. There were no formal exclusion criteria.

### CT scan protocols

CT exams were performed on multiple 64-slice to 128-slice CT systems (SOMATOM Sensation 64, Definition Flash, Definition Edge, Definition AS+; all Siemens Healthineers, Erlangen, Germany). Depending on the initial clinical indication of each scan, our cohort included non-contrast enhanced (non-CE) scans (*n* = 5,935, 32.5%) as well as contrast enhanced (CE) scans with different contrast phases [venous (*n* = 4,888, 26.8%), pulmonary-arterial (*n* = 4,203, 23.0%) and arterial (*n* = 2,233, 12.2%); of the arterial scans, *n* = 153 (0.8%) were ECG-triggered]. At our institution, we typically administer between 50 and 100 ml of contrast agent with flow rate between 2 and 5 ml/s depending on the specific scan protocol; this heterogeneity depended mainly on patients' weight and whether bolus tracking was used, for example in pulmonary-arterial phase. The thinnest soft tissue kernel of each exam was used for AIRad analysis (slice thickness = 0.6–1.0 mm, increment = 0.6 mm, resolution = 512 × 512 pixels).

### Deep learning algorithm

**Figure 2:**
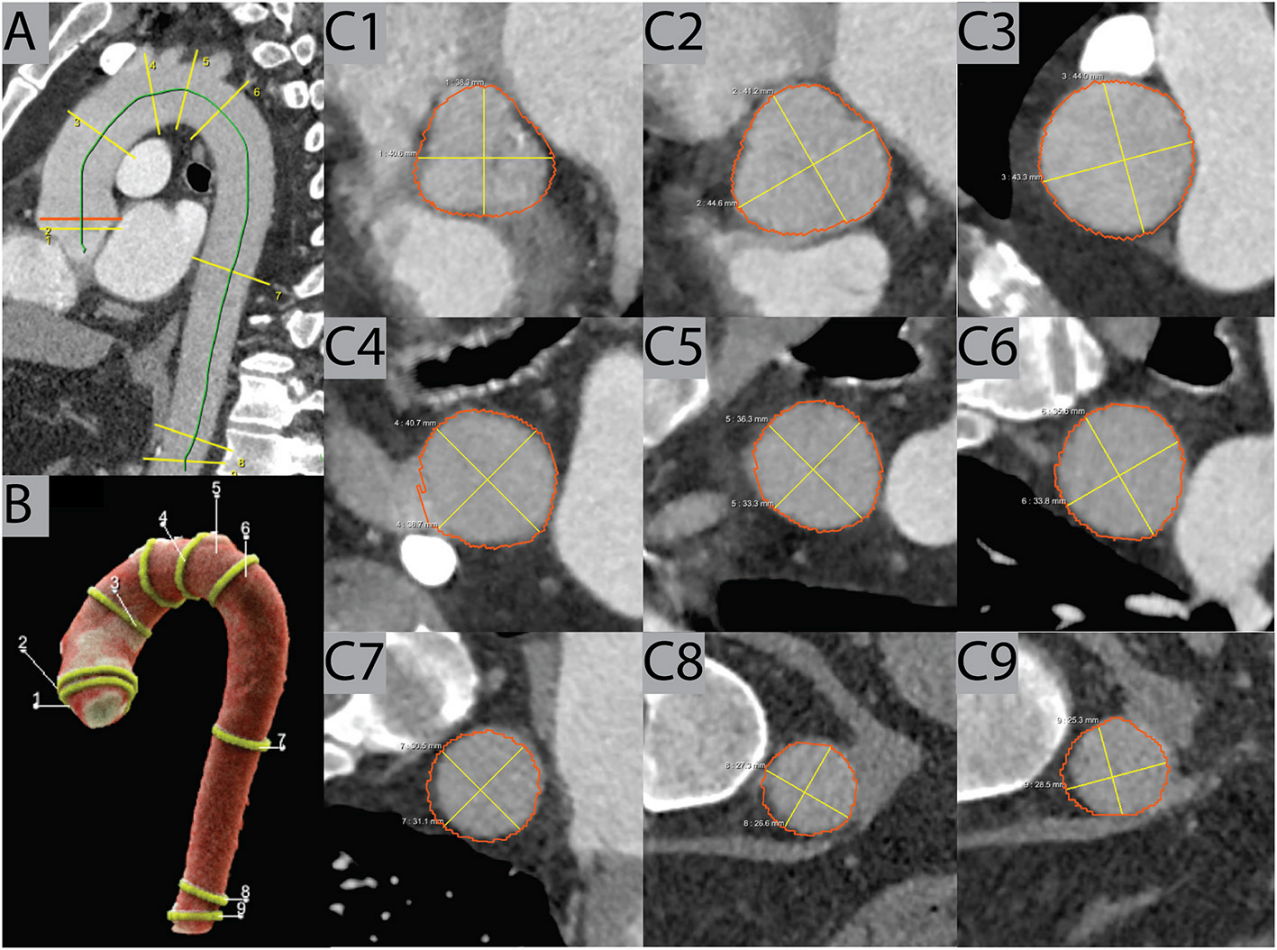
Example case. AIRad produced visual outputs consisting of a sagittal view on the thoracic aorta **(A)** and a 3D volume rendering **(B)** with all measured locations **(C1–C9)**. Furthermore, images of the measurements perpendicular to the centerline at each of the nine locations according to the AHA guidelines were also created, as seen in this example of an exam in pulmonary-arterial phase. AHA, American Heart Association.

AIRad measurements were performed by an in-house deployed prototype of AI-Rad Companion Chest CT (version 0.2.9.2, Siemens Healthineers, Forchheim, Germany). Its development was completely independent from this study, no scan analyzed in this study was used for training, validation or testing of AIRad. The underlying principle of AIRad was described elsewhere (10, 11, 14, 15). Briefly, it was trained on more than 10,000 data sets (CT data plus manual labeling of the six landmarks) for detection of aortic landmarks using deep reinforcement learning. Aortic segmentation was trained on more than 1,000 data sets (non-CE scans, different CE scans with and without ECG-triggering) using adversarial deep image-to-image network (14, 15). Training involved data sets from different vendors. AIRad fits a centerline into the segmented aorta which is followed by aortic diameter measurements according to the AHA guidelines (Figure 2) (4). At each location, the maximum in-plane diameter is reported. Visual output series are available in axial orientation as well as on a 3D volume rendering.

### Analysis workflow

The thinnest soft tissue kernel series per case was sent to the dedicated, on premise AIRad workstation which processed the cases one at a time. AIRad analyzed each case and reported the measurements for the locations as defined by the AHA (4). According to Mansour et al., we defined relevant dilatation as >45 mm for the proximal aorta (aortic sinus – AS, sinotubular junction – STJ, ascending aorta – AA, proximal arch – PA) and >40 mm for the distal aorta (mid arch – MA, distal arch – DA, mid descending aorta – MDA, distal descending aorta – DDA, and abdominal aorta - ABA); these cut-offs represent approximately an aneurysmatic dilatation per location according to the current AHA guidelines (4, 16).

After processing of all cases, a cardiovascular (CV) radiologist with 3 years' experience (MP) analyzed the measurement results regarding whether dilatation was found at least at one location for each case. If all measurements were below the respective cutoffs (classified as AIRad_negative), the case was counted as non-dilated and consistent with the radiologic report.

In case AIRad reported dilatation at least at one location (classified as AIRad_positive), all measurement locations of this case were reviewed in-detail by the radiologist using the visual output series. If dilatation was confirmed, the case was classified as true positive (TP). Otherwise, the case was counted as false positive (FP).

### Statistics

Data was organized Python (Python Software Foundation, Wilmington, USA). R (R Foundation for Statistical Computing, Vienna, Austria) was used for statistical analysis.

Discrete and continuous variables were tested for normal distribution and compared using either the student's *t*-test or Mann–Whitney *U*-test. Categorial variables were compared using Chi-square or binominal tests.

Two multivariable logistic regression models (MLRM) were created: the first to determine characteristics associated with AIRad_positive vs. AIRad_negative. The second MLRM was set up to determine characteristics associated with true positive (TP, AIRad_positive and dilatation confirmed) vs. FP (AIRad_positive but dilatation not confirmed) classification. In the MLRM, reference levels for categories were: non-CE scans, male sex, ECG-triggering and the numerical value of the diameter for the locations. A *p*-value <0.05 was defined as statistically significant.

## Results

### Baseline data

18,243/19,659 exams (92.8%) with normal aortic diameters according to the radiology reports were successfully processed by AIRad. The mean age was 62.3 ± 15.9 years, 8,330 were from female patients (45.7%) and the mean BMI was 25.6 ± 5.4 kg/m^2^. 13,620 scans were chest CT while the remaining 4,983 covered chest and abdomen. Please see Table 1 for baseline characteristics.

**Table 1:** Baseline characteristics for the entire cohort.

**Parameter**	
Number of scans	18,243
Age (years)	62.3 ± 15.9
Female sex	8,330 (45.7%)
Weight (kg)	74 ± 17.2
Height (m)	1.7 ± 0.1
BMI (kg/m^2^)	25.6 ± 5.4
**Exam type**
CT chest	13,260 (72.7%)
CT chest + abdomen	4,983 (27.3%)
**Contrast phase**
Non-contrast	5,935 (32.5%)
Arterial phase	2,233 (12.2%)
Pulmonary-arterial phase	4,203 (23%)
Venous phase	4,888 (26.8%)
Mixed contrast phase	768 (4.2%)
Other	216 (1.2%)
Scan with ECG-triggering	153 (0.8%)
DLP (mGycm)	330.4 ± 385.7

### Classification by AIRad

AIRad classified 17,239 cases (94.5%) as non-dilatated. On opposite, 1,004 cases (5.5%) were AIRad_positive, indicating a discrepancy between AIRad report and radiology report (Table 2). Mean age in this AIRad_positive cohort was 68.2 ± 12.4 years, 868 were male (86.5%). Of those cases, the majority were non-CE scans (*n* = 598, 59.6%), followed by venous phase (*n* = 193, 19.2%) and pulmonary-arterial phase (*n* = 114, 11.4%). The primary locations at which AIRad reported dilatation were the AS (*n* = 556, 55.4%), AA (*n* = 414, 41.2%), and STJ (*n* = 196, 19.5%); in 303 cases dilatation was reported at more than one location. An overview of mean diameters per location can be found in Table 3.

**Table 2:** Reviewed cohort, differences between true positive and false positive cases.

	**Total reviewed cases**	**True positive cases**	**False positive cases**	***p*-Value**
Number of scans	1,004	452 (45%)	552 (55%)	–
Age (years)	68.2 ± 12.4	68.3 ± 11.3	68.1 ± 13.2	0.85
Female sex	136	41 (30.1%)	95 (69.9%)	**0.001**
BMI (kg/m^2^)	27 ± 5.3	27.9 ± 5.5	26.2 ± 4.9	**0.001**
**Contrast phase**
Non-contrast	598	224 (37.5%)	374 (62.5%)	**0.001**
Arterial phase	59	27 (45.8%)	32 (54.2%)	1.0
Pulmonary-arterial phase	114	73 (64%)	41 (36%)	**0.001**
Venous phase	193	107 (55.4%)	86 (44.6%)	**0.005**
Mixed contrast phase	27	16 (59.3%)	11 (40.7%)	0.18
Other	13	5 (38.5%)	8 (61.5%)	0.78
Scan with ECG-triggering	9	1 (11.1%)	8 (88.9%)	0.09
DLP (mGycm)	343.2 ± 377	374.8 ± 369.2	317 ± 381.8	0.07
**Locations, AIRad_positive**
AS	556	256 (46%)	300 (54%)	0.35
STJ	196	120 (61.2%)	76 (38.8%)	**0.001**
AA	414	250 (60.4%)	164 (39.6%)	**0.001**
PA	48	27 (56.3%)	21 (43.8%)	0.13
MA	105	42 (40%)	63 (60%)	0.37
DA	68	32 (47.1%)	36 (52.9%)	0.77
MDA	27	7 (25.9%)	20 (74.1%)	0.07
DDA	40	14 (35%)	26 (65%)	0.28
ABA	34	5 (14.7%)	29 (85.3%)	**0.001**
**Number of positive locations**
1	612	244 (39.9%)	368 (60.1%)	**0.001**
2	198	106 (53.5%)	92 (46.5%)	**0.006**
3	79	53 (67.1%)	26 (32.9%)	**0.001**
4	19	14 (73.7%)	5 (26.3%)	**0.02**
5	7	5 (71.4%)	2 (28.6%)	0.26

Demographics, CT scan parameters and location-specific information on all cases reported as dilated by AIRad.

AA, ascending aorta; AS, aortic sinus; BMI, body mass index; DA, distal arch; DDA, distal descending aorta; DLP, dose length product; ECG, electrocardiogram; MA, mid arch; MDA, mid descending aorta; PA, proximal arch; STJ, sinotubular junction. Bold p values were statistically significant.

**Table 3:** Mean diameters per location in the true positive, false positive and non-dilated cohorts.

	**True positive cases**	**False positive cases**	**Non-dilated cases**
Number of cases	452	552	17,239
**Locations**
AS (mm)	44.42 ± 4.33	43.98 ± 4.75	35.20 ± 4.39
STJ (mm)	42.51 ± 4.03	40.74 ± 4.10	33.49 ± 4.01
AA (mm)	44.63 ± 3.55	42.78 ± 3.72	35.69 ± 4.14
PA (mm)	40.21 ± 3.27	39.02 ± 3.50	33.07 ± 3.82
MA (mm)	36.13 ± 3.06	35.62 ± 3.42	30.25 ± 3.49
DA (mm)	33.89 ± 3.91	33.76 ± 5.27	28.14 ± 3.34
MDA (mm)	31.67 ± 3.28	31.59 ± 4.67	26.54 ± 3.51
DDA (mm)	31.13 ± 3.95	31.40 ± 4.97	26.11 ± 3.75
ABA (mm)	29.37 ± 4.17	29.90 ± 5.76	25.12 ± 3.79

Mean diameters and standard deviation per location for true positive, false positive and negative cases.

AA, ascending aorta; ABA, abdominal aorta; AS, aortic sinus; DA, distal arch; DDA, distal descending aorta; MA, mid arch; MDA, mid descending aorta; PA, proximal arch; STJ, sinotubular junction.

### Detailed review of discrepant cases

**Figure 3:**
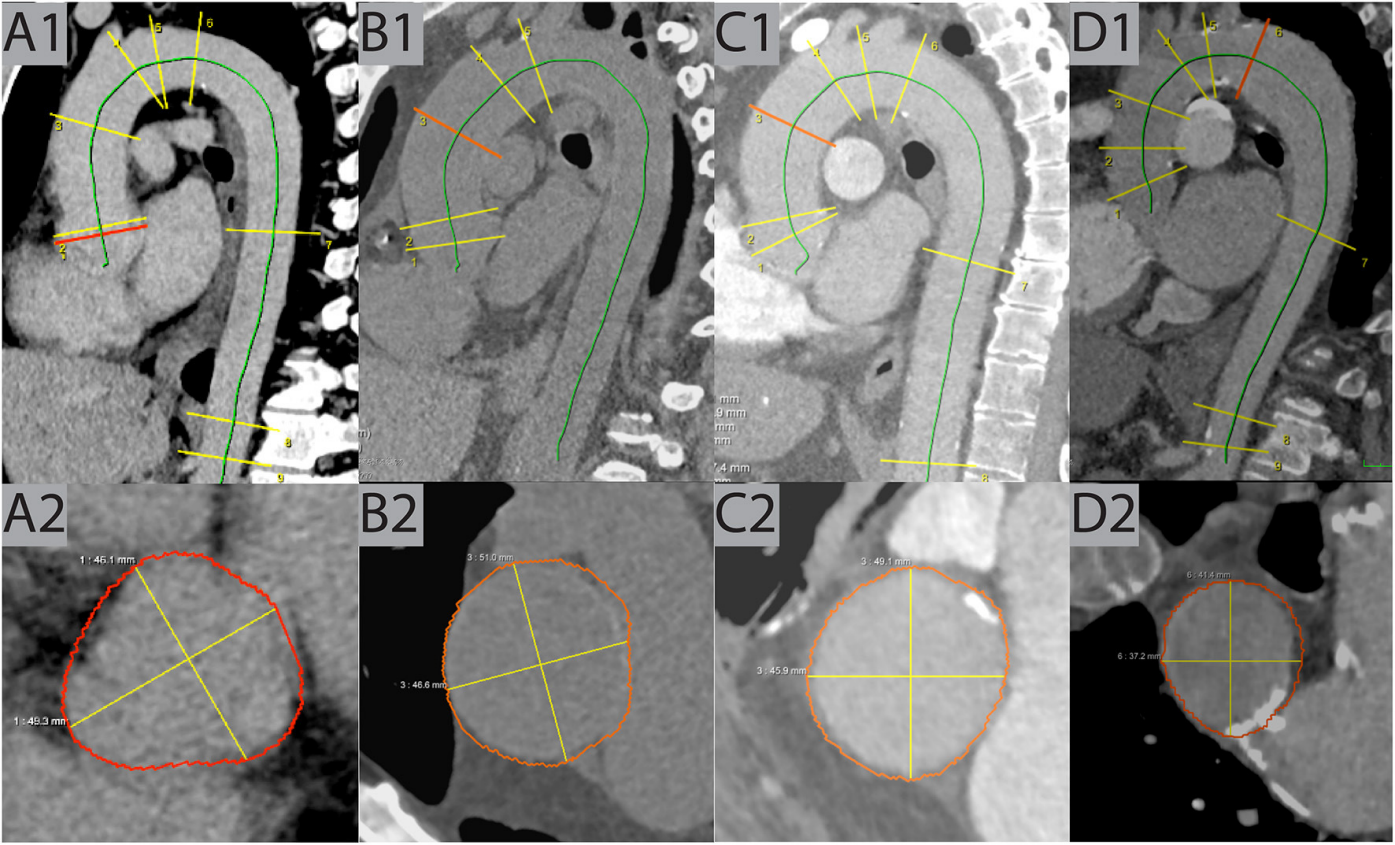
Examples of true positive cases. **(A)** This case shows dilatation of the AS (49 mm) in venous phase CT of a 57-year-old male patient detected by AIRad. **(B)** In this non-CE exam, dilatation of the AA (51 mm) was found in a 54-year-old male patient. **(C)** Similar to B but in pulmonary-arterial phase, AA dilatation (49 mm) in a 66-year-old male was revealed by AIRad. **(D)** Dilatation of the DA (41 mm) was identified by AIRad in a pulmonary-arterial phase CT of a 56-year-old female patient. AA, ascending aorta; AS, aortic sinus; CE, contrast enhanced; DA, distal arch.

**Figure 4:**
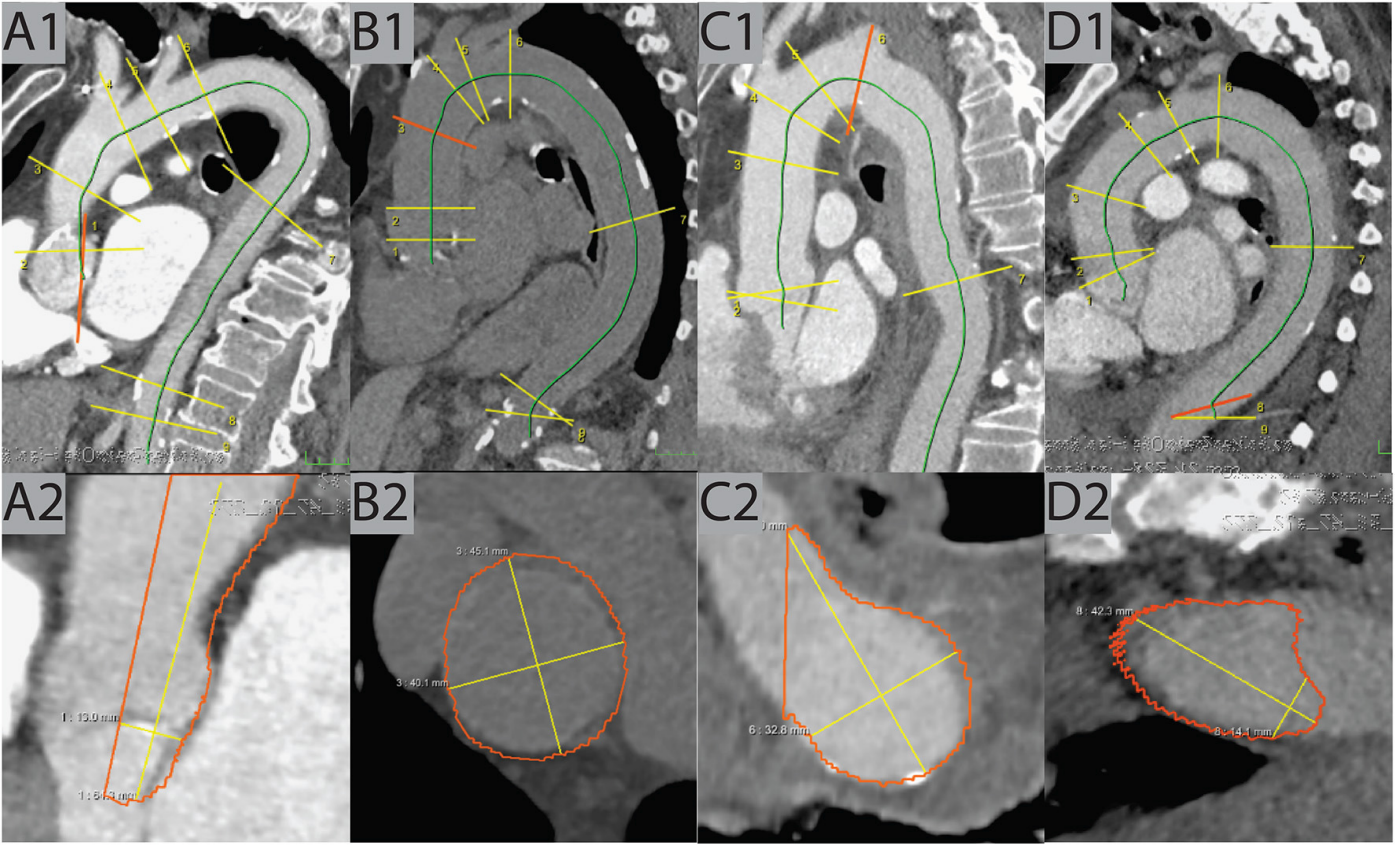
Examples of false positive cases. **(A)** Due to an error in centerline placement, the AS plane was tilted and falsely contoured, resulting in a false-high measurement in this pulmonary-arterial scan of a 93-year-old female patient. **(B)** In this non-CE scan of an 82-year-old female patient, the contouring at the AA location was too wide, resulting in a false-high measurement. **(C)** The CT in pulmonary-arterial phase of 75-year-old male patient showed an aberrant right subclavian arteria. This caused an error contouring the location of the DA, resulting in a false-high measurement. **(D)** The last two locations (DDA, ABA) in the pulmonary-arterial phase CT of an 85-year-old female patient were tilted caused by an erroneous centerline placement, resulting in a false-high measurement at DDA (42 mm). AA, ascending aorta; ABA, abdominal aorta; AS, aortic sinus; CE, contrast enhanced; DA, distal arch; DDA, distal descending aorta.

After in-detail review, dilatation was confirmed by the CV radiologists in 452 of 1,004 cases (45.0%) while assessment was FP in 552 cases (55.0%). Typical examples of TP and FP cases are shown in Figures 3, 4, respectively. In the TP subgroup, the mean diameter were high, especially at AA (44.63 ± 3.55 mm vs. FP subgroup: 42.78 ± 3.72 mm; Table 3). On opposite, the mean diameters for non-dilated cases were about 5–10 mm smaller.

In univariable comparisons, revealed the following factors to be associated with TP classification: male patients (*p* < 0.001) with a higher BMI (*p* < 0.001), in CE scans [*p* < 0.001, especially pulmonary-arterial phase (*p* < 0.001)], ECG-triggered scans (*p* < 0.001), dilatation at STJ (*p* = 0.002) and at AA (*p* < 0.001) or if TAD was reported at more than one location (*p* < 0.001). On opposite, dilatation reported at MDA (*p* < 0.001) and ABA (*p* < 0.001) was more likely FP. See Table 2.

Overall, AIRad classified 97.0% of all cases (17,691/18,243) correctly.

### Multivariable models

#### Characteristics of AIRad_positive vs. AIRad_negative prediction

Multivariable logistic regression models revealed that a higher BMI [Odds ratio (OR) = 1.09, *p* < 0.001], higher age (OR = 1.04, *p* < 0.001), and male sex (OR = 10.11, *p* < 0.001) were independently associated with AIRad_positive classification (Table 4). On opposite, a CE exam in arterial phase (OR = 0.31, *p* < 0.001), venous phase (OR = 0.43, *p* < 0.001) or pulmonary-arterial phase (OR = 0.40. *p* < 0.001) were independently associated with classification as AIRad_negative.

**Table 4:** MLRM Model 1 to differentiate between AIRad_positive vs. AIRad_negative.

	**Odds ratio**	**95% CI**	***p*-Value**
(Intercept)	0.000031	0–0.0002	**<0.001**
Age	1.04	1.03–1.04	**<0.001**
Male sex	10.11	7.05–15.06	**<0.001**
BMI	1.09	1.06–1.11	**<0.001**
**Contrast phase (vs. native)**
Arterial phase	0.31	0.19–0.46	**<0.001**
Pulmonary-arterial phase	0.4	0.29–0.55	**<0.001**
Venous phase	0.43	0.28–0.65	**<0.001**
Mixed contrast phase	0.6	0.34–0.99	0.06
Other	0.64	0.19–1.57	0.4
Scan with ECG-triggering	2.65	0.55–47.72	0.34
DLP	1	0.9997–1	0.73

Results from the MLRM Model 1 showed that cases reported as dilated (AIRad_positive) were more likely older, male patients with higher BMI. Furthermore, dilatation was less often reported in contrast-enhanced scans.

BMI, body mass index; DLP, dose length product; ECG, electrocardiogram; MLRM, multivariable logistic regression model. Bold p values were statistically significant.

#### Characteristics of true positive classification

Based on the second MLRM, the following parameters were independently associated with a TP classification by AIRad: dilatation found at STJ (OR = 1.09, *p* = 0.002) or AA (OR = 1.12, *p* < 0.001), higher BMI (OR = 1.05, *p* = 0.01), male sex (OR = 2.36, *p* = 0.003), any CE exams [mixed contrast phase (OR = 3.07, *p* < 0.02), pulmonary-arterial phase (OR = 2.92, *p* < 0.001), and venous phase (OR = 2.48, *p* = 0.01)], and when dilatation was found at more than one location (OR = 1.42, *p* = 0.008; Table 5). Of note, a smaller AS diameter was also associated with higher likelihood of TP classification by the AIRad (OR = 0.94, *p* = 0.02).

**Table 5:** MLRM 2 to differentiate between true positive vs. false positive cases.

	**Odds ratio**	**95% CI**	***p*-Value**
(Intercept)	0.000056	0–0.0041	**<0.001**
Age	1	0.98–1.01	0.6
Male sex	2.36	1.36–4.19	**<0.001**
BMI	1.05	1.01–1.08	**0.01**
**Contrast phase (vs. native)**
Arterial phase	2.08	0.97–4.45	0.06
Pulmonary-arterial phase	2.92	1.61–5.44	**<0.001**
Venous phase	2.48	1.21–5.13	**0.01**
Mixed contrast phase	3.07	1.21–8.16	**0.02**
Other	1.64	0.36–7.56	0.51
Scan with ECG-triggering	3.68	0.39–82.52	0.3
DLP	1	1–1	0.29
**Measurement location**
AS	0.94	0.9–0.99	**0.02**
STJ	1.09	1.03–1.15	**<0.001**
AA	1.12	1.06–1.19	**<0.001**
PA	1.04	0.96–1.13	0.35
MA	0.95	0.87–1.03	0.21
DA	1.01	0.96–1.06	0.73
Dilatation found at higher number of locations	1.42	1.1–1.84	**0.01**

MLRM 2 revealed that dilatation reported by AIRad was more likely to be correct in male patients with higher BMI, when contrast was administered as well as when dilatation was reported at AS, STJ, AA and at more than one location.

AA, ascending aorta; AS, aortic sinus; BMI, body mass index; DA, distal arch; DDA, distal descending aorta; DLP, dose length product; ECG, electrocardiogram; MA, mid arch; MDA, mid descending aorta; MLRM, multivariable logistic regression model; PA, proximal arch; STJ, sinotubular junction. Bold p values were statistically significant.

## Discussion

In this study, we evaluated the performance of AIRad as a secondary reading tool for detection of TAD in a large cohort of more than 18,000 scans with aortic diameters previously reported as *normal in size and caliber*. AIRad confirmed 17,239 scans as free of TAD and identified 452 scans with previously missed dilatation, resulting in correct assessment of 97% of all cases. Moreover, MLRM revealed that multiple parameters such as sex, BMI, CT contrast protocol, measurement location as well as number of reported TAD locations were independently associated with correct classification by AIRad.

Detection of TAD is important because it can lead to aortic dissection, rupture and death (17). In general, aortic aneurysms with diameters of >60 mm for the ascending or >70 mm for the descending aortic are associated with a rapid risk increase for a fatal outcome (18). Current guidelines therefore recommend that patients without high risk factors (such as Marfan Syndrome or bicuspid aortic valve) should undergo surgery if the aortic diameter is >55 mm; with risk factors present, the cut-off is even smaller (4). However, only few patients present with such enlarged aortas and since dilatation typically does not cause any symptoms, diagnosis tends to be incidental. In general, CT plays an important role in identifying patients with already dilatated aortas and diameter cut-offs are typically used to determine indication for follow-up imaging. These cut-offs were reduced over the years to currently 45 mm for the ascending and 40 mm for the descending aorta, respectively (16). They account for aortic growth rates between 0.1 and 0.2 mm/year (based on data from a large lung cancer screening cohort) and up to >1 mm/year in patients with underlying conditions (2, 19, 20). Therefore, patients with enlarged aortas should be identified and follow-up imaging for diameter assessment should be initiated.

Workload in the radiology department has dramatically increased over the past 15 years (9). In regards to TAD, a recent study suggested that the number of patients with TAD might actually be a lot higher than suspected (up to 40 times higher) which might lead to even more imaging studies (21). While DL-tools promise to assist radiologists, they actually add to the already increased workload (22). Recently, AIRad was successfully applied to ECG-triggered CT angiographies for the assessment of guideline-compliant, thoracic aortic diameters in small and moderate sized cohorts (up to few hundred exams) (10, 11). In this study, we extended AIRad application to a wider range of exams such as non-CE scans and also scans with different contrast phases. Overall, it correctly classified 17,691/18,243 (97%) of those exams with different protocols. In 1,004 of 18,243 cases, AIRad initially reported TAD of which 452 scans (45%) were confirmed by expert review. While the exact reason for missed diagnosis in these cases remains unknown, multiple types of errors for missed diagnosis are possible according to Kim and Mansfield (23): non-CE or e.g., pulmonary-arterial contrast phases imply that there was another, possibly urgent indication for these exams like infection or pulmonary embolism. This could have led to biases due to satisfaction of search, satisfaction of report and/or imaging technique. The candy-cane shape of the thoracic aorta was likely another source of error; diameter assessment and identification of dilatation at locations like the AS and STJ are more difficult due to their oblique position in regard to the standard multiplanar reformations. This is also relevant for parts of the aortic arch and can be considered an underreading error. Moreover, a technique error can in general be assumed when centerline analysis or double-oblique measurements were not used. This, however, is not feasible for every exam due to the time-consuming character of this task in the range of about 5 min per case (10). This time could potentially be saved using AIRad and highlights an advantage of an automatic DL tool for centerline-based aortic measurements which allows easy review *via* visual output series (24). While the number of TP findings was not high overall, about 2% of the whole cohort, this would have potentially influenced management of the 452 patients on an individual level, mainly by initiation of follow-up imaging. Therefore, we believe that future use of AIRad in chest CT could improve patient care.

AIRad's performance was further evaluated by MLRM: First, older, male patients with higher BMI were associated with higher likelihood of AIRad_positive classification while AIRad_negative classification was more likely in CE scans. More importantly, after in-detail review, the following factors were associated with correct, true positive classification: male sex, higher BMI, contrast protocol and location-specific characteristics. Men typically have larger aortic diameters than women and also a higher BMI (25, 26). These findings point to the general dilemma of absolute cut-offs which could be addressed by for example size or height adjusted diameter ratios with sex-specific adjustments in the future (27–29). Automatic diameter measurements could facilitate the implementation of such advancements.

Regarding contrast protocol, any type of contrast enhancement compared to non-CE scans was less likely to be labeled as AIRad_positive and also more likely to be TP after expert review. Assessment of the thoracic aorta is in general regarded to be easier after contrast administration for radiologists. This could partially explain why there were fewer cases labeled as AIRad_positive – they were less often missed in the radiology reports than in non-CE exams. However, AIRad still detected 228 CE scans which were TP after review; this highlights another advantage of a secondary reading by AIRad. In regards to non-CE exams, those represented the largest subgroup in our study with more than 5,900 scans total of which 598 were AIRad_positive. Of those 598 cases, 224 TP (37.5%) and 374 (62.5%) were FP after expert review, representing the lowest TP/FP ratio of all contrast subgroups. Diameter assessment in non-CE scans is generally more difficult for radiologists compared to CE scans. Our results suggest that this was true for AIRad as well. While the accuracy of AIRad in non-CE exams was lower compared to CE exams, it still detected 224 TP cases in non-CE exams. FP cases on the other hand could be easily identified by visual output series. In the future, re-training of AIRad with more non-CE training data could possibly hele improve its performance in non-CE exams.

The last important factor was related to TAD location. A larger diameter at STJ or AA was more likely associated with TP finding. This showed that our cohort had a high proportion of dilatation located in the proximal aorta which is the most common location of aortic dilatation in general (30). Interestingly, a smaller AS diameter was also associated with a TP finding. In fact, expert review revealed that TAD classification at the AS by AIRad was FP in more than half of all cases. A recent study performed aortic diameter measurements on non-CE scans using DL and a centerline approach and reported excellent results; but they excluded the locations of AS (and STJ) (31). However, this location is important since the aortic root (in combination of the ascending aorta) represent the primary sides of thoracic aortic aneurysms (30). As mentioned above, visual output series allow for easy assessment of TAD classification by AIRad. Lastly, the number of locations being classified as dilatated by AIRad was also independently associated with a TP finding. Radiologists probably missed those cases less often, therefore only few of these cases were included in our study, but AIRad still reliably detected those.

This is a retrospective, single-center study with limitations. All CT scans included were from one vendor, since AIRad was trained on CT data from multiple vendors, we believe that this should only have a minor effect on the results. Furthermore, a prototype version of AIRad was used in this study. This might explain failure to process 1,317/19,569 scans (6.7%), potentially because of erroneous landmark detection or segmentation which could be solved in future versions. The CT scans included in this study were preselected based on reported as *normal aortic diameters*; therefore, the study cohort does not represent the full spectrum of daily practice. We based this decision on the fact that we wanted to evaluate AIRad's performance as a secondary reading tool in a large number of scans and also non-ECG-triggered scans. But, this decision might limit the generalizability of our results. The ninth measurement location according to the AHA guidelines is in the abdominal aorta (at the level of the celiac trunk) which in general has lower diameter than the thoracic aorta. We still used the 40 mm cut-off since the DL tool was built to assess diameters in the thoracic aorta normal diameters in the descending aorta at the level of the diaphragm to not differ too much from that measurement location. Lastly, we did not systematically evaluate the more than 17,000 scans which were labeled AIRad_negative but considered them non-dilated. Overall, AIRad-based mean diameters in these cases were in accordance with previously reported normal ranges in a cohort in the same age range (25). However, we might have missed false negative cases with this approach. The immense effort required to verify all these cases and also our experience with AIRad being reliable in non-dilated cases was the basis for this decision.

## Conclusion

AIRad was successfully applied as a secondary reading tool for the assessment of TAD in a large cohort of CT chest exam with varying scan protocols. It correctly assessed the presence or absence of TAD in 17,691 (97.0%) of cases, including 452 cases with previously missed TAD. Our result thereby suggest the potential of AIRad to support the workflow by increasing report quality and efficiency.

## Data availability statement

The data analyzed in this study is subject to the following licenses/restrictions: data might be shared upon reasonable request and prior approval by the ethics committee. Requests to access these datasets should be directed at: MP, (maurice.pradella@usb.ch).

## Ethics statement

This retrospective study was approved by the local Ethics Committee (Ethikkommission Nordwest- und Zentralschweiz, ID: 2019-01053), and the need for informed consent was waived.

## Author contributions

MP: data analysis, statistical analysis and manuscript writing, study conception and design. RA: statistical analysis. JS, RK, and SR: technical development and manuscript editing. JC and SY: technical assistance and manuscript editing. GS, BS, JS, PB, and AS: study conception and design and manuscript editing. All authors contributed to the article and approved the submitted version.

## Conflict of interest

Authors JS, RK, and SR, are employees of Siemens Healthineers. Siemens Healthineers has a patent issued for this DL algorithm. The remaining authors declare that the research was conducted in the absence of any commercial or financial relationships that could be construed as a potential conflict of interest.

## Publisher's note

All claims expressed in this article are solely those of the authors and do not necessarily represent those of their affiliated organizations, or those of the publisher, the editors and the reviewers. Any product that may be evaluated in this article, or claim that may be made by its manufacturer, is not guaranteed or endorsed by the publisher.

## Abbreviations

AA: ascending aorta
ABA: abdominal aorta
AHA: American Heart Association
AI: artificial intelligence
AIRad: AI-Rad Companion
AS: aortic sinus
BMI: body mass index
CE: contrast enhanced
CT: computed tomography
CV: cardiovascular
DA: distal arch
DDA: distal descending aorta
DL: deep learning
ECG: electrocardiogram
FP: false positive
MA: mid arch
MDA: mid descending aorta
MLRM: multivariable logistic regression model
OR: odds ratio
PA: proximal arch
PACS: picture archiving and communication system
STJ: sinotubular junction
TAD: Thoracic aortic dilatation
TP: true positive
US: United States.
